# Evolving trends in epidemiology and natural history of cardiac amyloidosis: 30-year experience from a tertiary referral center for cardiomyopathies

**DOI:** 10.3389/fcvm.2022.1026440

**Published:** 2022-11-07

**Authors:** Aldostefano Porcari, Valentina Allegro, Riccardo Saro, Guerino Giuseppe Varrà, Linda Pagura, Maddalena Rossi, Andrea Lalario, Francesca Longo, Renata Korcova, Matteo Dal Ferro, Andrea Perkan, Franca Dore, Rossana Bussani, Giovanni Maria De Sabbata, Francesco Zaja, Marco Merlo, Gianfranco Sinagra

**Affiliations:** ^1^Department of Cardiovascular, Center for Diagnosis and Treatment of Cardiomyopathies, Azienda Sanitaria Universitaria Giuliano-Isontina (ASUGI), European Reference Network for Rare, Low Prevalence and Complex Diseases of the Heart-ERNGUARD-Heart, University of Trieste, Trieste, Italy; ^2^Department of Nuclear Medicine, Azienda Sanitaria Universitaria Giuliano-Isontina (ASUGI), University of Trieste, Trieste, Italy; ^3^Cardiothoracic Department, Center for Diagnosis and Treatment of Cardiomyopathies, Institute of Pathological Anatomy and Histology, Azienda Sanitaria Universitaria Giuliano-Isontina, University of Trieste, Trieste, Italy; ^4^Department of Hematology, Azienda Sanitaria Universitaria Giuliano-Isontina (ASUGI), Trieste, Italy; ^5^Department of Medical Science, University of Trieste, Trieste, Italy

**Keywords:** cardiac amyloidosis, epidemiology, diagnosis, prognostic stratification, non-invasive cardiac imaging

## Abstract

**Objective:**

Natural history of cardiac amyloidosis (CA) is poorly understood. We aimed to examine the changing mortality of different types of CA over a 30-year period.

**Patients and methods:**

Consecutive patients included in the “Trieste CA Registry” from January 1, 1990 through December 31, 2021 were divided into a historical cohort (diagnosed before 2016) and a contemporary cohort (diagnosed after 2016). Light chain (AL), transthyretin (ATTR) and other forms of CA were defined according to international recommendations. The primary and secondary outcome measures were all-cause mortality and cardiac death, respectively.

**Results:**

We enrolled 182 patients: 47.3% AL-CA, 44.5% ATTR-CA, 8.2% other etiologies. The number of patients diagnosed with AL and ATTR-CA progressively increased over time, mostly ATTR-CA patients (from 21% before 2016 to 67% after 2016) diagnosed non-invasively. The more consistent increase in event-rate was observed in the long-term (after 50 months) in ATTR-CA compared to the early increase in mortality in AL-CA. In the contemporary cohort, during a median follow up of 16 [4–30] months, ATTR-CA was associated with improved overall and cardiac survival compared to AL-CA. At multivariable analysis, ATTR-CA (HR 0.42, *p* = 0.03), eGFR (HR 0.98, *p* = 0.033) and ACE-inhibitor therapy (HR 0.24, *p* < 0.001) predicted overall survival in the contemporary cohort.

**Conclusion:**

Incidence and prevalence rates of ATTR-CA and, to a less extent, of AL-CA have been increasing over time, with significant improvements in 2-year survival of ATTR-CA patients from the contemporary cohort. Reaching an early diagnosis and starting disease-modifying treatments will improve long-term survival in CA.

## Introduction

Although previously considered as a rare and orphan disease, cardiac amyloidosis (CA) is increasingly recognized as frequent cause of heart failure (HF) and mortality in recent years (1). Light chain (AL) amyloidosis has an estimated prevalence of 1–2 in every 100.000 subjects (2). Although the exact epidemiological figure of transthyretin (ATTR) amyloidosis is still under scrutiny, this condition is more prevalent than traditionally thought, being reported in the heart of 25–40% of unselected adults > 75 years (3, 4). In contemporary years, the identification of populations at higher prevalence of CA (5–10) and major advances in non-invasive techniques such as cardiac magnetic resonance imaging and bone scintigraphy for the non-biopsy diagnosis of disease have led to a considerable increase in CA recognition, redefining the paradigm of cardiac involvement in amyloidosis (11, 12). The impact of these diagnostic advancements on clinical profiles at presentation and on the natural history of patients with CA has played a crucial role in ATTR-CA (13), but a comparison between AL and ATTR-CA has not been addressed so far. This is a crucial knowledge-gap to be covered as AL and ATTR-CA are completely different forms of amyloidosis in terms of pathophysiology, management and treatment options. AL amyloidosis is a treatable condition with different chemotherapy regimens and autologous stem cell transplantation, while ATTR-CA has become treatable after the first disease-modifying treatment has been tested in the ATTR-ACT trial in 2018 (14–16).

Therefore, we aimed to analyze the trends in epidemiology and natural history of patients with CA diagnosed over a 30-year period at a tertiary referral center for cardiomyopathies.

## Materials and methods

This is a single-center, retrospective, observational study performed at the Cardiovascular Department, Cattinara University Hospital, Azienda Sanitaria Universitaria Giuliano-Isontina (ASUGI) and University of Trieste, Trieste, Italy. The local Regional Institutional Review Board approved the study (identifier 43_2009). The study was conducted according to the Declaration of Helsinki and informed consent was obtained under the institutional review board policies of the hospital administration.

### Study population and definitions

Consecutive patients diagnosed with CA at the Cardiovascular Department, Azienda Sanitaria Universitaria Giuliano-Isontina (ASUGI), between January 1st, 1990 and December 31th, 2021 from the Trieste CA Registry were included in the study population and their data were retrospectively reviewed. The diagnosis of CA was made in presence of “invasive” or “non-invasive” criteria, according to the position statement on diagnosis and treatment of CA of the European Society of Cardiology (12). In detail, ATTR-CA was diagnosed in presence of a Perugini grade 2 or 3 myocardial uptake at cardiac scintigraphy with bone tracers and absence of monoclonal protein at urine and serum tests. Histological confirmation of amyloid deposition in the heart by endomyocardial biopsy (EMB) or in other affected tissues was obtained in all patients suspected of AL-CA with monoclonal proteins and in those not fulfilling non-invasive criteria (12). For the purpose of the study, patients were divided into an “historical cohort” (enrolled < 2016) and a “contemporary cohort” (enrolled ≥ 2016) according to the validation of non-invasive diagnostic work-up for ATTR-CA (17). None of the patients with ATTR-CA received disease-modifying treatments during the study period.

### Characterization of patients

Patients’ baseline was set at the time of CA diagnosis and clinical data performed within 1 month were collected from electronic medical records, including all the following: (i) clinical history and examination, (ii) electrocardiogram (ECG), (iii) echocardiography, and, (iv) blood tests. ECG and echocardiographic images stored on our electronic database were systematically reviewed offline for this specific study by three cardiologists (A.P., M.M., L.P.), blinded to patients’ outcome. Twelve-lead ECG was performed using standard equipment and retrospectively reviewed for heart rate, rhythm, QRS voltage, depolarization and repolarization abnormalities. Low voltages were defined as a QRS amplitude ≤ 0.5 mV in all limb leads or ≤ 1 mV in all precordial leads (18). All echocardiographic parameters were measured according to standard international definitions (19). Left ventricle (LV) volumes and LV ejection fraction (LVEF) were calculated using the Simpson’s biplane method. Restrictive filling pattern (RFP) was defined as E-wave deceleration time < 120 ms or ≤ 150 ms in the presence of E/A ≥ 2. Right ventricle (RV) systolic dysfunction was defined as a tricuspid annular plane systolic excursion (TAPSE) < 17 mm and/or fractional area contraction (FAC) < 35% (19). The presence and severity of valve disease was defined according to current recommendations (20).

Cardiac scintigraphy with technetium pyrophosphate (99mTc-PYP) was performed with acquisition of planar and single photon emission computed tomography. A semi-quantitative score for the LV was obtained based on results of planar images as described by Perugini (21). The cameras and acquisition protocol used at our Institution is shown in Supplementary Table 1.

Histological evaluation of cardiac and extra-cardiac tissues was performed by the chief of our Institute of Pathological Anatomy and Histology (R.B.) with a specific expertise in the cardiovascular (CV) field, according to the standards and definitions proposed by the Committee of the Society for Cardiovascular Pathology and the Association for European Cardiovascular Pathology (22). In particular, histological sections were stained with hematoxylin and eosin (H&E) and Congo red, carefully analyzed for the presence of amyloid infiltration in the myocardium and vessels and evaluated under a polarized light microscope (23). Immunohistochemistry with kappa and lambda light chains antibodies, anti-TTR antibodies, anti-apolipoprotein AI and anti-serum amyloid A antibodies was performed on the most representative sample for each patient to characterize the amyloid deposits (4).

### Outcome

The primary outcome of the study was all-cause mortality. The secondary outcome measure was cardiac death. The events were collected from the dedicated electronic databases of our center and, if needed, from patients’ general practitioners and/or telephone contacts with patients and their relatives. At our institution, protocols of coroner referral and post-mortem analysis were constant over time. Events were independently assessed by three cardiologists (G.G.V., R.S, F.L.), blinded to patients’ characteristics.

### Statistical analysis

Descriptive statistics were measured as median with interquartile range (IQR) [25°; 75°] for continuous variables as data were not normally distributed according to the results of Kolmogorov-Smirnov test; categorical variables were expressed as absolute numbers and percentages. Differences between groups were evaluated using Mann–Whitney test for continuous variables, while Chi square (χ2) or Fisher’s exact test were used for dichotomous variables. The Kaplan-Meier method was used to estimate overall survival, and the log rank test was used to compare the curves. In the case of secondary end points, to account for the presence of competing risks, cumulative incidence curves were estimated and compared using appropriate methods (24). Univariable and multivariable analyses were performed for the primary and secondary study outcomes in patients from the contemporary cohort. Each variable was evaluated at univariable cause-specific Cox regression and, when a *p*-value < 0.1 was found, was included into a multivariable Cox model. The number of events was taken into account to estimate an adjusted HR with an event per variable (epv) ratio of 10. The end of follow-up was set at 31th December, 2021. We defined a *p*-value < 0.05 as statistically significant. All statistical analyses were performed using IBM SPSS Statistics 24.0 package (New York, NY) statistical software version 20 and R (R Foundation for Statistical Computing, Vienna, Austria)^1^, packages “cmprsk” and “crrSC.”

## Results

### Increasing prevalence of cardiac amyloidosis over time

**FIGURE 1:**
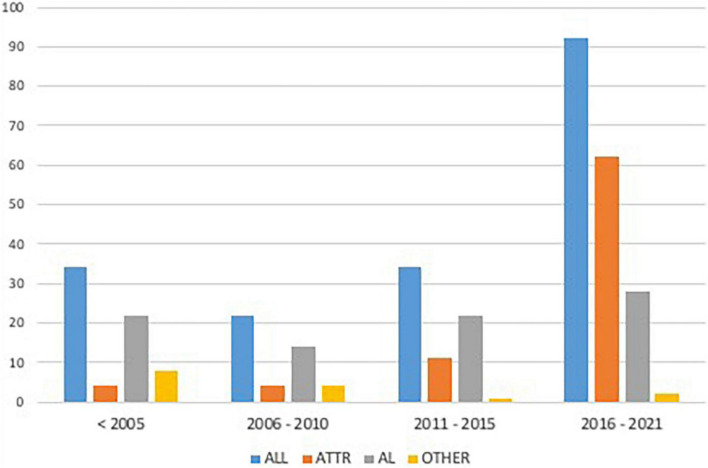
Enrolment rate of the study population according to different periods. AL, Light Chain Amyloidosis; ATTR, Transthyretin Amyloidosis.

The study population included 182 patients diagnosed with CA: 49.5% (*n* = 90) from the “historical cohort” and 50.5% (*n* = 92) from the “contemporary cohort.” CA was related to the following etiologies: 47.3% (*n* = 86) AL, 44.5% (*n* = 81) ATTR, and 8.2% (*n* = 15) other etiologies (5 dialysis-related amyloidosis, 10 undetermined etiologies) (Figure 1).

**FIGURE 2:**
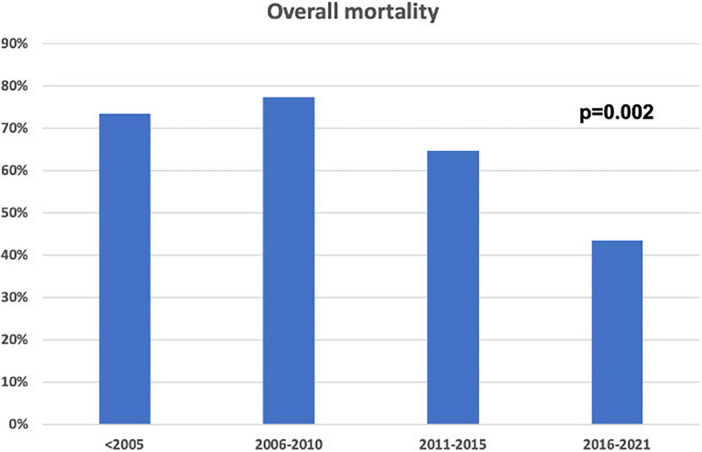
Overall mortality in the study population according to enrolment periods.

Over time, the consistent increase in non-invasive CA diagnosis by cardiac scintigraphy (59.8 vs. 3.3%, *p* < 0.001) was paralleled by a decrease in the number of CA diagnosed by biopsy (96.7 vs. 40.2%, *p* < 0.001), which still was adopted in a significant quota of cases in the contemporary cohort (i.e., mostly for AL-CA confirmation). Mortality in patients with CA diagnosed in recent years was lower compared to the past (*p* = 0.002, Figure 2). Chemotherapy regimens used in patients with AL-CA are shown in Supplementary Table 2.

### Characterization of cardiac amyloidosis over time

Table 1 shows the baseline characteristics of the study population according to the enrolment period. All patients presented with HF. Compared to the historical cohort, patients of the contemporary cohort were predominantly males (75 vs. 58.9%, *p* = 0.021), more affected by ATTR-CA (67.4 vs. 21.1%, *p* < 0.001), presented less frequently in NYHA ≥ 3 (36.3 vs. 53.5%, *p* = 0.021), and had greater rates of AF (53.8 vs. 19.1%, *p* < 0.001), LVEF < 50% (36.8 vs. 22.1%, *p* = 0.034) and aortic valve stenosis (any degree of severity) (21.1 vs. 6%, *p* = 0.005) (Table 1).

**TABLE 1:** Baseline characteristics of the study population according to the enrolment period.

	Available (*n*)	Study population (*n* = 182)	< 2016 (*n* = 90)	> 2016 (*n* = 92)	*P*-value
Age, y	**182**	**73 (63–80)**	**70 (58–77)**	**77 (69–82)**	**<0.001**
Male	**182**	**122 (67%)**	**53 (58.9%)**	**69 (75%)**	**0.021**
AL amyloidosis	**182**	**86 (47.3%)**	**58 (64.4%)**	**28 (30.4%)**	**0.001**
ATTR amyloidosis	**182**	**81 (44.5%)**	**19 (21.1%)**	**62 (67.4%)**	**0.001**
Other etiologies	**182**	**15 (8.2%)**	**13 (14.4%)**	**2 (2.2%)**	**0.001**
Biopsy-proven diagnosis	**182**	**124 (68.1%)**	**87 (96.7%)**	**37 (40.2%)**	**<0.001**
Scintigraphy-proven diagnosis	**182**	**58 (32%)**	**3 (3.3%)**	**55 (59.8%)**	**<0.001**
NYHA ≥ 3	**177**	**79 (44.6%)**	**46 (53.5%)**	**33 (36.3%)**	**0.021**
Systolic BP, mmHg	169	120 (110–140)	120 (110–140)	120 (110–140)	0.176
Syncope	182	19 (10.4%)	12 (13.3%)	7 (7.6%)	0.20
Hypertension	**182**	**99 (54.4%)**	**40 (44.4%)**	**59 (64.1%)**	**0.008**
eGFR < 60 mL/min	151	77 (51%)	33 (50%)	42 (45.6%)	0.83
eGFR, mL/min	151	50 (36–75)	59 (34–75)	59 (40–76)	0.484
IHD	182	12 (6.6%)	5 (5.6%)	7 (7.6%)	0.58
History of AF	**180**	**66 (36.7%)**	**17 (19.1%)**	**49 (53.8%)**	**<0.001**
Carpal tunnel	**181**	**44 (24.3%)**	**6 (6.7%)**	**38 (41.3%)**	**<0.001**

		**Medications**			

BBs	171	82 (48%)	36 43.4%)	46 (52.3%)	0.244
ACEi/ARBs	171	86 (50.3%)	36 (43.4%)	50 (56.8%)	0.079
Diuretics	171	143 (83.6%)	69 (83.1%)	74 (84.1%)	0.866
MRAs	171	68 (39.8%)	30 (36.1%)	38 (43.2%)	0.347

		**Electrocardiography**			

Rhythm at baseline	182				0.145
Sinus rhythm		109 (59.9%)	60 (66.7%)	49 (53.3%)	
AF		61 (33.7%)	24 (26.7%)	37 (40.2%)	
PM		12 (6.6%)	6 (6.7%)	6 (6.5%)	
HR, bpm	145	75 (65–88)	76 (68–90)	70 (65–85)	0.110
RBBB	182	30 (16.5%)	10 (11.1%)	20 (21.7%)	0.053
LBBB	182	27 (14.8%)	10 (11.1%)	17 (18.5%)	0.162
LFAB	182	31 (17%)	12 (13.3%)	19 (20.7%)	0.189
LVH	182	21 (11.5%)	9 (10%)	12 (13%)	0.521
Q wave	182	55 (30.2%)	32 (35.6%)	23 (25%)	0.121
Low QRS voltages	182	64 (35.2%)	37 (41.1%)	27 (29.3%)	0.097

		**Echocardiography**			

LVEDVi, mL/m2	**155**	**42 (34–55)**	**38 (30–51)**	**45 (38–57)**	**0.002**
IVS, mm	**174**	**16 (14–19)**	**15 (14–18)**	**17 (15–20)**	**0.016**
PW, mm	167	14 (12–16)	14 (12–16)	14 (12–17)	0.347
LVEF	**167**	**55% (47–63)**	**57% (51–65)**	**53% (41–62)**	**0.021**
LVEF < 50%	**173**	**51 (29.5%)**	**19 (22.1%)**	**32 (36.8%)**	**0.034**
E/E’	**112**	**20 (14–27)**	**20 (11–23)**	**21.5 (16–28)**	**0.035**
RFP	124	61 (49.2%)	32 (47.8%)	29 (50.9%)	0.729
LA diameter, mm	142	44 (39–50)	43 (39–49)	46 (40–51)	0.101
RA area, cm2	131	23 (18–26)	22 (18–26)	23 (19–27)	0.466
RV dysfunction	165	91 (55.2%)	46 (56.1%)	45 (54.2%)	0.808
Biventricular dysfunction	161	40 (24.8%)	17 (21.3%)	23 (28.4%)	0.294
Moderate-severe MR	149	30 (20.1%)	14 (20%)	16 (20.3%)	0.969
Aortic stenosis	**159**	**21 (13.2%)**	**5 (6%)**	**16 (21.1%)**	**0.005**
RV hypertrophy	174	69 (39.7%)	31 (36%)	38 (43.3%)	0.336
Thickened IAS	**174**	**26 (14.9%)**	**8 (9.3%)**	**18 (20.5%)**	**0.039**
Pericardial effusion	174	69 (39.7%)	37 (43%)	32 (36.4%)	0.369

ACEi, Angiotensin-converting enzyme inhibitor; AF, Atrial Fibrillation; AL, Amyloid light chain; ARBs, Angiotensin II Receptor Blockers; ATTR, Transthyretin Amyloidosis; BBs, Beta Blockers; BP, Blood Pressure; eGFR, estimated Glomerular Filtration Rate; HR, Heart Rate; IAS, Interatrial Septum; IHD, Ischemic Heart Disease; IVS, Interventricular septum; LA, Left Atrium; LBBB, Left Bundle Branch Block; LFAB, Left Fascicular Anterior Block; LVEDVi, Left Ventricular End Diastolic Volume index; LVEF, Left Ventricular Ejection Fraction; LVH, Left Ventricular Hypertrophy; MR, Mitral regurgitation; MRAs, Mineralocorticoid Receptors Antagonists; NYHA, New York Heart Association; PM, Pacemaker; PW, Posterior Wall; RA, Right Atrium; RBBB, Right Bundle Branch Block; RFP, Restrictive Filling Pattern; RV, Right Ventricle. Bold identifies parameters with *p*-value <0.05.

Compared to the historical ATTR-CA cohort, ATTR-CA patients in the recent cohort had a similar age at diagnosis (mean age 78 vs. 80 years, *p* = 0.174), higher frequency of history of AF (67.2 vs. 38.9%, *p* = 0.030) and increased E/E’ ratio (21 vs. 17, *p* = 0.046) (Supplementary Table 3). Of note, none of the patients before 2016 presented with NYHA 1, while none of the patients after 2016 presented with NYHA 4. Similar cardiological characteristics were found for AL-CA patients.

### Prognostic implications of amyloidosis type in the contemporary and historical cohorts

**FIGURE 3:**
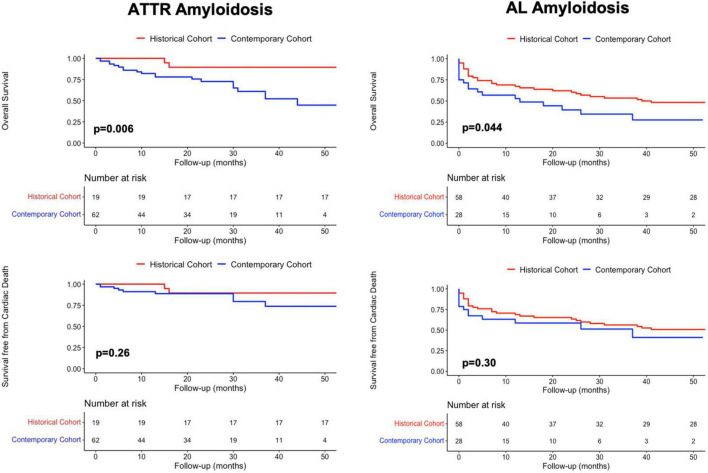
Kaplan-Meier survival analysis in patients from the historical and the contemporary cohort with **(Left column)** ATTR amyloidosis and **(Right column)** AL amyloidosis for overall mortality and cardiac death. AL, Light Chain Amyloidosis; ATTR, Transthyretin Amyloidosis.

**FIGURE 4:**
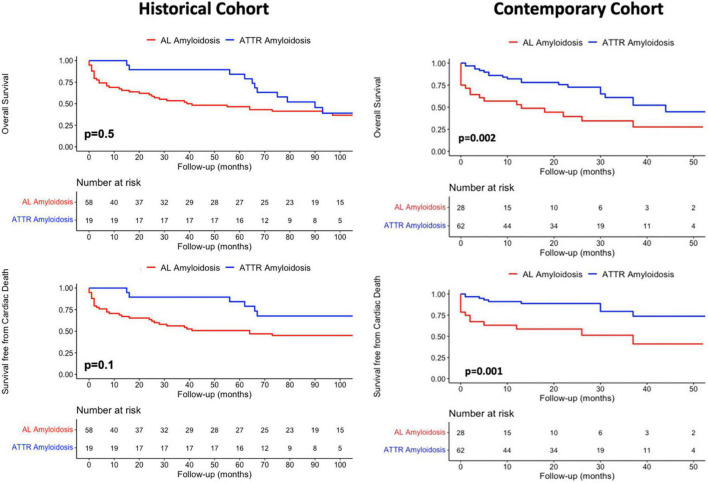
Kaplan-Meier survival analysis for **(Top row)** overall mortality and competing risk analysis for **(Bottom row)** cardiac death according to amyloidosis etiology in the historical and contemporary cohorts. AL, Light Chain Amyloidosis; ATTR, Transthyretin Amyloidosis.

Overall mortality of patients with ATTR-CA was significantly higher in the contemporary compared to the historical cohort (*p* = 0.006), while no difference was found in cardiac mortality rates (*p* = 0.26) (Figure 3, left). AL-CA patients from the contemporary cohort had higher frequency of all-cause death (*p* = 0.044) and similar rates of cardiac mortality (*p* = 0.3) compared to those from the historical cohort (Figure 3, right). During a median follow up of 65 [8–118] months, at survival analysis, patients with AL-CA and ATTR-CA from the historical cohort had similar rates of all-cause death (67 and 74% respectively, *p* = 0.5) and cardiac death (57 and 42% respectively, *p* = 0.1) (Figure 4, left). In the historical cohort, an early separation of the survival curves was observed due to a higher event-rate in patients with AL-CA that was paralleled over time by a progressive increase in the event-rate in patients with ATTR-CA, with similar long-term outcome in both types of amyloidosis. In the contemporary cohort, over a median follow up of 16 [4–30] months, ATTR-CA was associated with more favorable outcome compared to AL-CA, with an observed overall survival of 80 vs. 40% at 24 months (*p* = 0.002; Figure 4, right). At multivariable analysis using significant covariates emerged at univariable analysis, in patients from the contemporary cohort, ATTR-CA (overall survival HR 0.42, *p* = 0.03; cardiac death HR 0.39, *p* = 0.042), ACE-inhibitor therapy (overall survival HR 0.24, *p* < 0.001; cardiac death HR 0.25, *p* = 0.07) and eGFR (overall survival HR 0.98, *p* = 0.033; cardiac death HR 0.96, *p* = 0.041) were associated with both study outcomes (Table 2).

**TABLE 2:** Univariable and multivariable analyses for overall mortality and cardiac death in the contemporary cohort.

Parameters	Univariable analysis		Multivariable analysis (OM)		Multivariable analysis (CD)	
	HR (95% CI)	*P*-value	HR (95% CI)	*P*-value	HR (95% CI)	*P*-value
Age at diagnosis, for every year increase	1.001 (0.96–1.03)	0.953				
Male sex	**0.49 (0.25–0.95)**	**0.035**	0.62 (0.29–1.34)	0.08	0.78 (0.26–2.30)	0.66
Scintigraphy vs. biopsy	**0.23 (0.12–0.46)**	**<0.001**				
ATTR vs. AL	**0.39 (0.20–0.73)**	**0.004**	**0.42 (0.18–0.93)**	**0.038**	**0.39 (0.15–0.96)**	**0.042**
History of AF	0.76 (0.41–1.42)	0.394				
Syncope	2.44 (0.87–5.75)	0.126				
Hypertension	1.08 (0.56–2.07)	0.812				
SBP, mmHg	0.98 (0.96–1.007)	0.173				
Carpal tunnel	0.54 (0.27–1.07)	0.151				
NYHA ≥ 3	1.76 (0.94–3.29)	0.110				
eGFR < 60 ml/min	1.15 (0.61–2.18)	0.655				
eGFR, ml/min	0.98 (0.97–1.001)	0.07	**0.98 (0.97–0.99)**	**0.033**	**0.96 (0.94–0.98)**	**0.041**
Low QRS voltages	1.81 (0.95–3.45)	0.123				
BBs	0.58 (0.30–1.11)	0.104				
ACEi/ARBs	**0.24 (0.12–0.48)**	**<0.001**	**0.24 (0.11–0.50)**	**<0.001**	**0.25 (0.09–0.69)**	**0.007**
IVS, per every mm increase	1.006 (0.94–1.07)	0.866				
LVEF, per every% increase	1.004 (0.98–1.02)	0.748				
LVEF < 50%	1.02 (0.52–1.99)	0.943				
E/E’, for every point increase	0.99 (0.96–1.03)	0.903				
RFP	0.47 (0.21–1.05)	0.112				
LA diameter, mm	0.99 (0.96–1.02)	0.858				
RV dysfunction	0.89 (0.46–1.73)	0.741				
Biventricular dysfunction	0.69 (0.31–1.52)	0.360				
Moderate-severe MR	1.37 (0.61–3.04)	0.438				
Aortic Stenosis	1.41 (0.68–2.92)	0.355				
Pericardial effusion	0.99 (0.50–1.93)	0.979				

ACEi, Angiotensin-converting enzyme inhibitor; AF, Atrial Fibrillation; AL, Amyloid light chain; ARBs, Angiotensin II Receptor Blockers; ATTR, Transthyretin Amyloidosis; BBs, Beta Blockers; CD, Cardiac Death; eGFR, estimated Glomerular Filtration Rate; IVS, Interventricular septum; LA, Left Atrium; LVEF, Left Ventricular Ejection Fraction; LVH, Left Ventricular Hypertrophy; MR, Mitral regurgitation; NYHA, New York Heart Association; OM, Overall Mortality; RFP, Restrictive Filling Pattern; RV, Right Ventricle, SBP, Systolic Blood Pressure. Bold identifies parameters with *p*-value <0.05.

## Discussion

The present study describes the changes in epidemiology, clinical profiles and natural history of CA over the last 30 years in a third-level referral Center for cardiomyopathies. To the best of our knowledge, this is the first report including comprehensive cardiological characterization and investigating baseline predictors for global outcomes and cardiovascular outcomes in a combined cohort of AL and ATTR-CA patients across different time periods.

The major findings of the present study are that (a) the number of patients diagnosed with CA progressively increased over time, mostly related to ATTR-CA; (b) CA patients from the contemporary cohort presented with less symptomatic HF compared to those from the historical cohort; (c) although an early increase in the event-rate was found in AL-CA, long-term survival rates among ATTR-CA and AL-CA were similar in the historical cohort, while, in the contemporary cohort, ATTR-CA was associated with reduced all-cause mortality and cardiac mortality compared to AL-CA; and, (d) in the contemporary cohort, ATTR-CA, higher eGFR and therapy with ACE-inhibitors were associated with a more favorable global and cardiovascular outcome. Rather than a change in natural history of the disease, we believe that the observed increase in incidence and prevalence of CA is likely related to a number of factors including (1) heightened awareness of disease, (2) recognition of clinical and instrumental red-flags and subgroups of patients at higher risk of CA, and (3) development of non-invasive criteria for the diagnosis and specific treatments (25, 26).

### The evolving epidemiology of cardiac amyloidosis

In the present analysis, the number of patients diagnosed with AL and ATTR-CA progressively increased over time. In recent years, bone scintigraphy has become the predominant mode of diagnosis for CA, resulting in a significant increase of patients diagnosed with ATTR-CA and, to a lesser extent, with AL-CA (Figure 1) (5–8, 12). Of note, our results suggest that the improved diagnostic yield has led to the identification of more patients with early as well as advanced CA (Table 1). Notably, current approach to CA resulted in increased recognition of AL-CA as patients with suspicion of ATTR-CA undergo a comprehensive assessment, including search for monoclonal proteins in urine and serum, thus leading to identification of otherwise undiagnosed patients with AL-CA (27). However, the diagnostic approach to AL-CA has not significantly changed over the years as reflected in similar cardiac phenotype at presentation among AL-CA patients from the historical and contemporary cohort These findings are in line with recent data from referral centers for amyloidosis reporting evidence of substantially greater recognition of both AL and ATTR amyloidosis (28). Although AL amyloidosis still remains the most common type of amyloidosis in national referral centers, accounting for 55% of all cases (29), differences in the structure of national health systems, referral patterns and populations’ ethnicities may explain this discrepancy among different centers (1, 30).

### The natural history of transthyretin and light chain-cardiac amyloidosis across ages

Patients with CA from the historical cohort had very poor outcomes regardless of type of amyloidosis, reasonably related to recognition of disease in late stages and lack of effective therapies. In our cohort, the natural history of AL-CA was characterized by an early increase in all-cause mortality rate, especially in the months following initiation of chemotherapy (Figure 4). Interestingly, in ATTR-CA patients from the historical cohort, the consistent increase in event-rate was observed in the long-term (after 50 months; Figure 4, top, left), supporting the possibility to effectively change the natural history of disease with disease-modifying therapies. In the contemporary cohort, ATTR-CA was associated with more favorable global and cardiovascular outcomes compared to AL-CA, reflecting the major advances in earlier diagnosis and treatment, especially for ATTR amyloidosis (Figure 4). In 2016, a landmark study by Gillmore et al. (17) paved the way for the clinical application of bone scintigraphy for the non-invasive diagnosis of ATTR-CA, demonstrating that the positive predictive value of a moderate-high myocardial uptake approaches 100% in the absence of a monoclonal protein in serum and urine, thus limiting the need for EMB to selected cases (31, 32). Broadening the diagnostic horizon of CA, predominantly ATTR-CA (9), resulted in recognition of more patients in different stages of cardiac disease and in improved overall survival in the contemporary cohort compared to AL-CA patients (Figure 3). The observed lower overall survival of patients with ATTR-CA and AL-CA from the contemporary compared to the historical cohort, in spite of similar rates of cardiac death, results from the increasing competing risks of non-cardiac death that is typical of elderly patients (25) (Figure 3). In the past, many patients with CA, especially ATTR amyloidosis, were not recognized or diagnosed at post-mortem examination, as reflected by the lower absolute number of ATTR-CA patients in the historical compared to the contemporary cohort (Table 1). Our results are in line with recent reports from nationwide studies that shows a progressive reduction in overall mortality of CA over years (30).

### Tools for prognostic stratification in the contemporary era

In this study, the type of amyloidosis (AL vs. ATTR amyloidosis), renal function and tolerability of ACE-inhibitor therapy were associated with a better global and cardiac outcome in the contemporary cohort (Table 2). The prognostic role of renal function has been largely investigated and this parameter is included in validated prognostic scores (26, 33).

Although CA confers increased risk of mortality and morbidity, the more favorable natural history of ATTR-CA compared to AL-CA was expected based on the heterogeneity and severity of organ involvement found in patients presenting with AL amyloidosis in clinical practice, whose survival is largely dependent on the tolerability and efficacy of chemotherapy. These findings further underline that AL and ATTR amyloidosis are 2 different diseases. The goal of early initiation of specific treatment would be to obtain an increase in survival with net and persistent separation of the curves in the contemporary cohort rather than a progressive decline in survival observed in untreated patient from the historical cohort (Figure 3). Of note, the association between ACE-inhibitor therapy and survival is of particular interest. In our opinion, patients tolerating these drugs might have a less advanced systemic and cardiac amyloid burden rather than having direct survival advantages from this treatment. According to recent studies, ACE-inhibitors and beta-blockers might be safely prescribed in CA, starting from low doses, then slowly up-titrated with frequently re-evaluation of treatment tolerance (34, 35). Dedicated studies are required to understand whether these drugs have a prognostic impact in patients with HF due to amyloidotic etiology.

## Limitations

This is a single-center retrospective study conducted in a third-level referral center for the diagnosis and management of cardiomyopathies. Therefore, the expertise of our Center in this field is a potential bias to consider. NT-proBNP and troponin could not be systematically included in the analysis as they were routinely evaluated from 2018 on (for NT-proBNP) or because of a change in the assay sensitivity over time (high-sensitive troponin evaluated from 2019 on). CMR data were not available in this analysis. Etiology-specific prognostic predictors on multivariable analysis could not be investigated in the present study because of a limited number of events in the contemporary cohort; however, this is an important issue to investigate in future dedicated studies. Finally, at our institution, ATTR-CA patients underwent systematically genetic testing for transthyretin mutations after 2016 and all of them were diagnosed with wild-type form.

## Conclusion

Recent years have been characterized by an exponential increase in incidence and prevalence rates of CA, especially ATTR amyloidosis. In the modern era, patients with ATTR-CA have more favorable global and cardiovascular outcome compared to those with AL-CA. In the contemporary cohort, a diagnosis of ATTR-CA, renal function and ACE-inhibitor therapy at presentation were associated with a more favorable global and cardiovascular outcome.

## Data availability statement

The raw data supporting the conclusions of this article will be made available by the authors, without undue reservation.

## Ethics statement

The studies involving human participants were reviewed and approved by the Regional Institutional Review Board approved the study (identifier 43_2009). The patients/participants provided their written informed consent to participate in this study.

## Author contributions

APo, MM, and GS contributed to conception and design of the study. VA, RS, GV, LP, MR, AL, and FL organized the database. APo performed the statistical analysis. APo, VA, and MM wrote the first draft of the manuscript. MD, RK, APe, FD, RB, GD, and FZ wrote sections of the manuscript. All authors contributed to manuscript revision, read, and approved the submitted version.

## Footnotes

1
https://www.r-project.org/
