# Supplementary material for: Evolving trends in epidemiology and natural history of cardiac amyloidosis: 30-year experience from a tertiary referral center for cardiomyopathies
Source: Front Cardiovasc Med. 2022 Nov 7;9:1026440. doi: 10.3389/fcvm.2022.1026440 (PMC9677956; doi:10.3389/fcvm.2022.1026440)
Supplement: Supplementary file 1 [file Data_Sheet_1.docx]

**Supplementary Table 1. Technical characteristics, camera bone tracer and acquisition protocol adopted by the Department of Nuclear Medicine in Trieste.**

| **Parameters** | **Characteristics** |
| --- | --- |
| **Acquisition machine** | Symbia SPECT/TC T2 Siemens |
| **Tracer molecule** | PYP |
| **Mean dose per exam** | 370 MBq |
| **Planar images (matrix)** | 256x256 |
| **SPECT images (matrix)** | 64x64 |
| **Collimator** | LEHR |
| **Acquisition after injection** | 60 and 80 min |
| **Elaboration software for planar images** | Hermes NM Processing |
| **Elaboration software for SPECT images** | Hermes Hybrid Reconstruction Cardiology  Hermes Hybrid Recon |

**Legend:** LEHR, low-energy high-resolution; SPECT, single-photon emission computed tomography.

**Supplementary Table 2. Chemotherapy regimens in patients with AL-CA.**

| **Chemotherapy** | **N. of patients treated** |
| --- | --- |
| Bortezomib based | 26 |
| Melphalan based | 39 |
| IMID based | 6 |
| Daratumumab based | 1* |
| ASCT | 6 |
| Untreated | 9^ |
| Total | 86 |

Legend: AL-CA, Light Chain Cardiac Amyloidosis; ASCT, Autologous Stem Cell Transplantation; IMID, Immunomodulatory Drugs. *Daratumumab had evidence for AL amyloidosis from clinical randomized trials from July 2021. ^ Patients in this group belong to the historical cohort, presented with end-stage AL-CA foreclosing treatment and died shortly after diagnosis.

**Supplementary Table 3. Baseline characteristic of ATTR and AL-CA from the historical and the contemporary cohort.**

|  | **AL-CA <2016 (n=58)** | **AL-CA >2016 (n=28)** | **p value** | **ATTR-CA <2016 (n=19)** | **ATTR-CA >2016 (n=62)** | **p value** |
| --- | --- | --- | --- | --- | --- | --- |
| **Age,** | 67 (58-76) | 70 (59-73) | 0.8 | 78 (70-80) | 80 (74-83) | 0.17 |
| **Male** | 31 (53%) | 18 (64%) | 0.2 | 14 (73.7%) | 50 (80.6%) | 0.5 |
| **Hypertension** | 23 (40%) | 13 (46%) | 0.3 | 11 (58%) | 44 (71%) | 0.28 |
| **eGFR<60 ml/min** | 23 (55%) | 15 (58%) | 0.5 | 7 (39%) | 28 (49%) | 0.4 |
| **Syncope** | 6 (10%) | 3 (11%) | 0.6 | **6 (31.6%)** | **4 (6.5%)** | **0.004** |
| **NYHA >2** | **35 (61%)** | **11 (41%)** | **0.04** | 8 (44.4%) | 22 (36%) | 0.49 |
| **History of AF** | 8 (14%) | 7 (25%) | 0.16 | **7 (39%)** | **41 (67%)** | **0.03** |
| **LBBB** | 7 (12%) | 4 (14.3%) | 0.5 | 2 (10.5%) | 12 (19.4%) | 0.37 |
| **RBBB** | 6 (10%) | 6 (21%) | 0.14 | 2 (10.5%) | 14 (22.6%) | 0.24 |
| **IVS, mm** | 15 (14-19) | 15 (14-18) | 0.9 | 15 (12-21) | 18 (15-20) | 0.13 |
| **E/E’** | 20 (13-27) | 22 (12-30) | 0.4 | **17 (10-20)** | **21 (16-27)** | **0.046** |
| **LVEF<50%** | 14 (24%) | 8 (30%) | 0.3 | 4 (25%) | 23 (40%) | 0.21 |
| **RFP** | 24 (52%) | 10 (48%) | 0.4 | 5 (42%) | 18 (53%) | 0.37 |
| **RV dysfunction** | 32 (58%) | 13 (50%) | 0.3 | 8 (50%) | 32 (57%) | 0.4 |
| **Aortic Stenosis** | 3 (5%) | 2 (8%) | 0.4 | 1 (6%) | 12 (24%) | 0.09 |
| **BBs** | 22 (39%) | 11 (44%) | 0.7 | 8 (47%) | 34 (56%) | 0.3 |
| **ACEi/ARBs** | 20 (36%) | 9 (36%) | 0.5 | 10 (59%) | 39 (64%) | 0.7 |

**Legend:** ACEi: Angiotensin-converting enzyme inhibitor; AF: Atrial Fibrillation; AL: Amyloid light chain; ARBs: Angiotensin II Receptor Blockers; ATTR: Transthyretin Amyloidosis; BBs: Beta Blockers; CA: Cardiac Amyloidosis; eGFR: estimated Glomerular Filtration Rate; IVS: Interventricular septum; LBBB: Left Bundle Branch Block; LVEF: Left Ventricular Ejection Fraction; NYHA: New York Heart Association; RBBB: Right Bundle Branch Block; RFP: Restrictive Filling Pattern; RV: Right Ventricle.
